# Central Neurocytoma in a Teenager, a Rare Cause of Hemiplegia, and a Diagnostic Dilemma in a Resource-Poor Setting

**DOI:** 10.1155/2024/4514981

**Published:** 2024-03-05

**Authors:** Kofi Ulzen-Appiah, Kafui P. Akakpo

**Affiliations:** Department of Pathology, School of Medical Sciences, University of Cape Coast, Cape Coast Teaching Hospital, Cape Coast, Ghana

## Abstract

**Background:**

Central neurocytoma is a benign intraventricular neuroectodermal tumor most often arising in the lateral ventricles. Due to the location of this tumor, common signs and symptoms include obstructive hydrocephalus, recurrent headache, visual impairment, nausea, and vomiting. Central neurocytoma and intraventricular oligodendroglioma share similar gross features and cellular and architectural morphology, which may pose a diagnostic challenge in a poor resource setting. Immunohistochemical neuronal stains are useful for the two tumors in our setting. *Report*. An 18-year-old male patient presented with a 1-year history of right-sided weakness, recurrent seizures, and sudden loss of consciousness. The patient showed signs of increased intracranial pressure, but an antemortem CT or MRI scan could not be done to determine the underlying cause, due to lack of availability and poor prognosis of the patient at the time of presentation. An autopsy revealed a well-demarcated solid cystic, gritty intraventricular tumor causing obstructive hydrocephalus, with associated dilated ventricles and severe cerebral edema. Postmortem histopathological examination of the tumor confirmed central neurocytoma.

**Conclusion:**

Central neurocytoma (CN) is an uncommon cause of intracranial space occupying lesion (ICSOL) in the teenage age group in our setting. Central neurocytoma and intraventricular oligodendroglioma share similar radiologic and histomorphological features. Immunohistochemical evaluation with neuronal markers is essential in these two tumors, as they have different prognoses and surgical and treatment outcomes.

## 1. Introduction

Intracranial space occupying lesions can cause severe debility. The clinical presentation of intracranial space-occupying lesions (ICSOL) varies depending on the location of the tumor and may develop over a period. In our setting, infectious organisms such as tuberculosis and other fungal and protozoan organisms are common causes of ICSOL and thus may cause delays in the clinical diagnosis of ICSOL that are of neuronal or neuroglial and glial in origin [1]. Radiological appearance and location may help differentiate between such space-occupying lesions and are a necessary adjunct to clinical, histopathological examination and diagnosis of ICSOL [2, 3].

Central neurocytomas (CNs) are rare tumors in young adults, comprising about 0.25%-0.5% of all intracranial tumors [1, 4]. The majority of cases are present in the third and fourth decades of life [1, 4]. Tumor incidence shows no significant gender preponderance [4]. CNs are mostly located in the lateral ventricles next to the foramen of Monroe and were initially thought to arise from precursor cells of the subependymal plate of the ventricular system and the circumventricular organs [5]. According to the recent World Health Organization (WHO) Classification of Tumors of the Central Nervous system (5^th^ edition-2021), CNs are Grade II tumors classified under neuronal and mixed neuroglial tumors, and the cell of origin is now thought to be unclear [3, 6]. Rarely, CNs arise in the fourth ventricle and other extraventricular sites [4].

Due to their location, affected patients present with signs and symptoms of increased intracranial pressure from obstructive hydrocephalus, and these include recurrent headaches, visual disturbances, nausea, and vomiting [2]. Radiologically, CNs are hyperattenuating compared to white matter, calcifications are present and usually punctate with large tumors having cystic areas [2, 7].

Gross total resection (GTR) with complete tumor removal is the treatment of choice for CN, and radiation therapy (RT) is indicated when complete resection is not attained or possible. CNs have a good prognosis after complete tumor resection with excellent long-term survival (5-year survival 81%) [8].

We present a teenager with symptoms and signs of ICSOL and review the available literature.

## 2. Case Report

An 18-year-old male patient presented to our facility with a one-year history of right-sided weakness, recurrent seizures, and sudden loss of consciousness of 3 days duration. Before this, the patient had recurrent headaches and impaired vision. According to the mother, about a year ago, she noticed her son had developed a weakness on the right side with associated slurred speech and deviation of the mouth to the right. His condition became worse with associated seizures and impaired consciousness.

Neurologic examination revealed reduced consciousness (Glasgow coma score of 8/15). On inspection, there were no abnormal movements, no abnormal posture, and no induced or spontaneous fasciculations. There was a normal tone in the left upper limb but increased in the right upper limb. Power could not be assessed objectively due to his reduced consciousness level. There was increased reflexes (knee and ankle reflexes). The plantar response on the right foot was extensor. Modalities of sensation and coordination could not be assessed also as a result of his reduced consciousness. Pupils were equal with sluggish response to light bilaterally but worse on the right with a size of 3 mm. Cardiovascular examination revealed a blood pressure and pulse rate of 100/70 mmHg and 70 b/min, respectively. Heart sounds were present and normal. The apex beat was in the left 5^th^ intercostal space, midclavicular line, and normal. Respiratory examination revealed no chest wall deformities, a central trachea resonant percussion note, and markedly reduced normal breath sounds bilaterally in all lung zones.

The patient was managed clinically for a recurrent cerebrovascular accident complicated by seizures with differential diagnoses of subarachnoid hemorrhage, electrolyte imbalance, meningitis, and intracranial space-occupying lesion in this order. Unfortunately, no MRI or CT imaging and laboratory studies were done to establish a definitive diagnosis before his demise. The patient died after 2 hours on admission, as a result of worsening increased intracranial pressure leading to brainstem herniation and loss of cardiorespiratory function.

**Figure 1:**
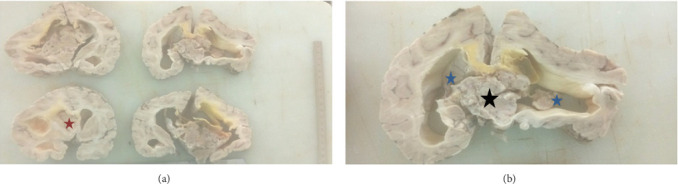
Serial sections of the fixed brain show flattening of the gyri, obliteration of the sulci, and dilation of the lateral ventricles ((b) blue stars), consistent with increased intracranial pressure from the intraventricular grey solid cystic tumor with gritty consistency effacing the septum pellucidum into the third ventricle inferiorly (black star), and extending anteriorly into the left lateral ventricle ((a) red star).

An autopsy revealed, a brain weighing (2,000 g), diffuse flattening of gyri, and obliteration of the sulci suggestive of cerebral edema/increased intracranial pressure postfixation serial coronal sections of the brain, showed a well-demarcated gritty grey nodular tumor measuring 7 × 6 × 5 cm, with punctate dark brown zones and microcystic areas effacing the septum pellucidum and occupying the third ventricle and extending into the left lateral ventricle (Figure 1). The lateral ventricles were dilated with a midline shift to the left as well as grooving of the cerebellar tonsils. The lungs were both heavy (600 g each); the parenchyma of both lungs showed severe congestion, foci of hemorrhage, and mild edema. The heart was normal, as were the kidneys. The adrenal glands were also normal. All other organ systems examined were grossly normal.

**Figure 2:**
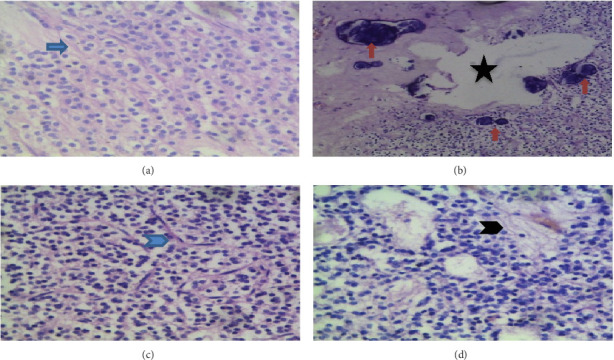
Showing (H&E 100x) a tumor composed of cords and single files of monotonous round cells, with clear cytoplasm and a central nucleus with stippled (salt and pepper) chromatin ((a) blue arrow). There is an intervening fibrillary matrix and a thin fine-branching capillary network ((c) blue arrowhead). (b) Areas with microcysts (black star) and microcalcifications (orange arrows). (d) A rare pseudoperivascular rosette (pineocytomatous rosette)-like formation (pseudo palisading of lesional cells around a central vessel) (black arrowhead).

Microscopic sections of the tumor showed cords and single files of monotonous round cells with distinct cell borders, clear cytoplasm, and central nucleus with stippled (salt and pepper) chromatin, giving a characteristic fried egg appearance (perinuclear halo). The stroma is loose and fibrillary, containing thin, fine branching capillaries (chicken wire) with areas of microcalcification and microcysts. Occasional pineocytomatous/pseudoperivascular rosettes are seen; however, cellular atypia, mitotic figures, endothelial proliferation, and necrosis are absent (atypical features) (Figure 2).

**Figure 3:**
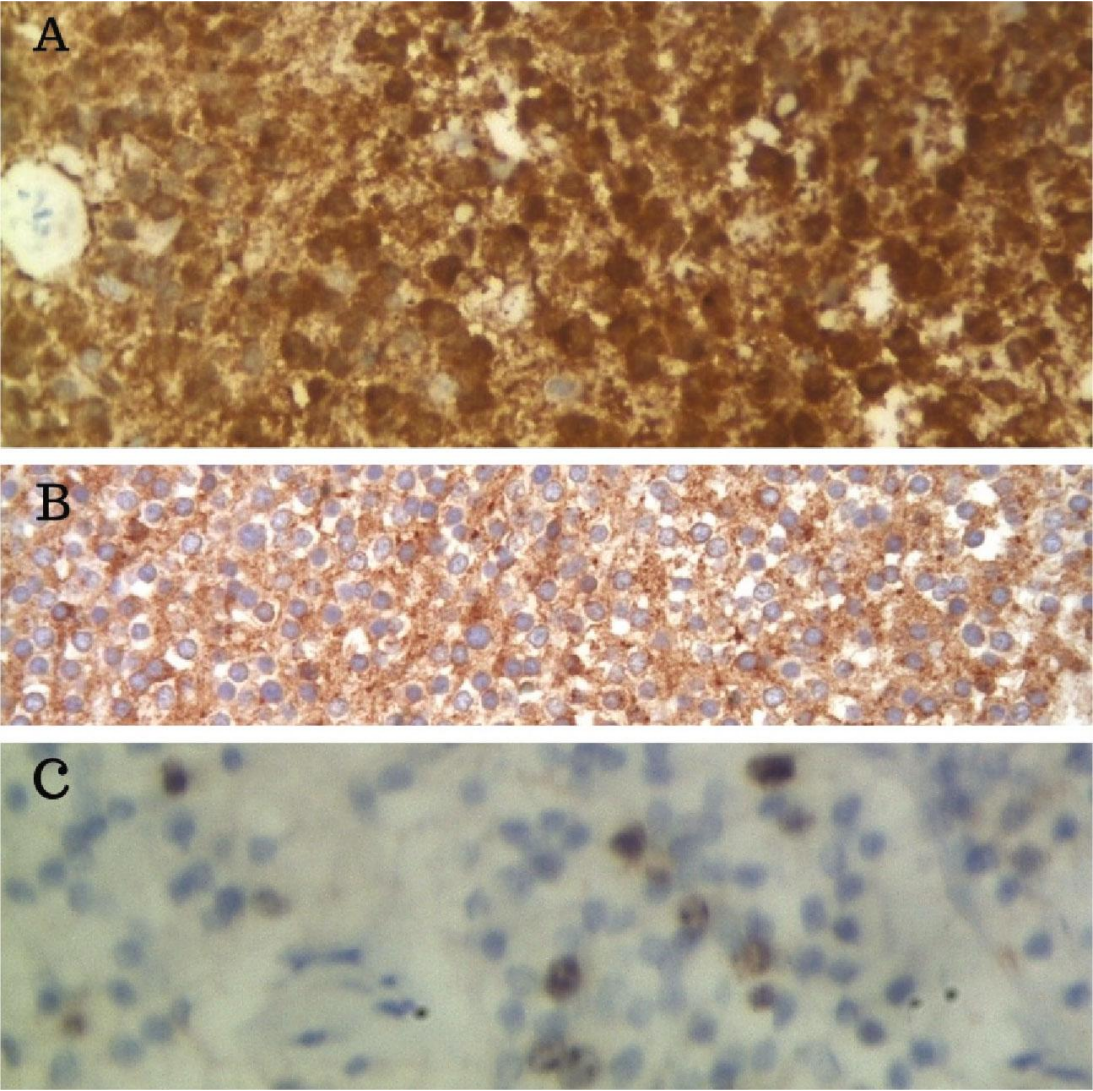
(A) Diffuse intense cytoplasmic staining for neuron-specific enolase (NSE) (400x); (B) diffuse granular staining of the fibrillary matrix (neuropil areas) for synaptophysin (SYN) (400x); (C) occasional nuclear reactivity for Ki-67 (400x).

Immunohistochemical stains were positive for neuronal markers (intense diffuse cytoplasmic staining for neuron-specific enolase) and diffuse granular staining of intercellular fibrillary matrix and perivascular areas for SYN (synaptophysin) and low Ki-67 (<1%) (Figure 3).

## 3. Discussion

CN was first described in 1982 by Hassoun et al. [9]. They described it as a benign tumor composed of a relatively mature neuronal population of neurocytes evidenced by clear vesicles, microtubules, synaptic structures, and abundant cytoplasmic processes on electron microscopy [9]. Since the first description of CN by Hassoun et al. [9] to date, to our knowledge, two cases of the tumor have been reported in Africa [10, 11].

Characteristically, CNs are located in the supratentorial ventricular region, 50% in the lateral ventricles, and 13% in both lateral and third ventricles, and the solitary third ventricle CNs account for only 3% [4]. There are reported cases of extra ventricular locations [12]. Our patient's tumor was supratentorial, arising from the septum pellucidum and occupying the third ventricle extending into the left lateral ventricle. A similar case has been reported in a 54-year-old male with CN who presented with an intracranial bleed and obstructive hydrocephalus [13].

Patients usually present with features of increased intracranial pressure due to tumor location obstructing the normal flow of cerebrospinal fluid [4]. Affected patients usually present with headaches, associated nausea and vomiting, and visual impairment due to papilloedema, or abducens nerve palsy (causing diplopia) [2]. In some cases, intraventricular hemorrhage may occur [14]. Our patient presented with right-sided weakness recurrent seizures and sudden loss of consciousness which is unusual for most of the reported cases of CN. Such an atypical presentation has been reported in a fourteen-year-old male patient who presented with progressive abnormal involuntary tremulous movement of the left hemi body (dystonic tremor) of three-year duration [15]. The unusual or atypical clinical presentation of right-sided weakness may be explained by the large size of the intraventricular mass (7 cm across) causing midline shift to the left and the extension of the tumor into the left lateral ventricle producing pressure/mass effect on the left frontoparietal lobar parenchyma and subsequent ischemia. CNs have been reported to have a median size of 4.2 cm in diameter [16]. The enlarging mass results in obstruction of the third ventricle and accumulation of cerebrospinal fluid within the lateral ventricles dilating the ventricular system, which in turn leads to obstructive hydrocephalus. According to the Monro-Kellie doctrine, the adult skull is fixed, and nonyielding and the sum of volumes of the brain, cerebrospinal fluid, and intracranial blood are constant [17]. To maintain normal intracranial pressure, an increase in any of the compartments will result in a decrease in the other compartments to maintain equilibrium until autoregulatory mechanisms are lost [18]. The enlarging mass together with obstructive hydrocephalus both lead to the accumulation of cerebrospinal fluid within the ventricular system and the pressure or mass effect of the left cerebral hemisphere, leading to the unusual clinical presentation.

It has been reported that CNs are well-demarcated grey nodular solid cystic tumors with gritty consistency [2], and differential diagnoses for intraventricular tumors of pediatric and adult age groups include ependymoma, subependymal giant cell astrocytoma (SEGA), intraventricular meningioma, choroid plexus papilloma, and intraventricular oligodendroglioma (IO) [15]. The septum pellucidum is lined by glial cells and residual neuronal precursor cells from which CN and IO may arise. The ependymal cells and cuboidal cells lining the ventricles may give rise to ependymoma and choroid plexus papilloma, respectively. Arachnoid cap cells, which make up arachnoid granulations, may become trapped within the choroid plexus during embryonic development, and these cells may give rise to meningiomas [4]. Intraventricular tumors with calcification in the pediatric and young adult age group include CN and IO. Oligodendroglioma may also arise within the body of the lateral ventricle but characteristically has ill-defined and infiltrative borders. Our patient had a well-demarcated intraventricular solid tumor with cystic and punctate hemorrhagic areas and gritty consistency. From the gross appearance and location of the lesion, we favored a CN with a differential diagnosis of IO as CN is much more common. Ependymomas usually lack cystic and calcified components [19].

On computed tomography scans, CNs are isointense to slightly hyperdense with smooth margins and small low-density areas [2]. The tumor is usually attached to the septum pellucidum, and calcification is present in approximately 50% of all CNs, which is characteristically punctate or coarse with multiple small cysts [2].

Magnetic resonance imaging shows CN as a well-circumscribed heterogeneous mass with lobulated margins. The tumor is usually isointense or slightly hypointense to gray matter on T1-weighted images and hyperintense on T2 images [20]. It demonstrates mild to moderate enhancement with intravenous gadolinium administration [20]. IO tends to occur within the body of the lateral ventricle, is infiltrative, and has large and irregular calcifications [7]. Unfortunately, we were unable to perform CT or MRI scans on our patient to characterize the radiologic appearance of the ICSOL.

Microscopically, CN and IO are very similar, although the cells of CN have neuronal features of stippled (salt and pepper) chromatin [9]. Both lesions on haematoxylin- and eosin-stained slides show monotonous round cells with clear halo and central nuclei (fried egg appearance), thin fine branching (chicken wire) vasculature, and calcifications (Figure 2). These two tumors are often misdiagnosed based on their cellular morphology and surrounding stroma [21]. However, the identification of pineocytomatous rosettes distinguishes CN from IO in most cases [2]. Our case showed cellular morphology consistent with neuronal features and pineocytomatous rosettes (Figure 2(d)).

Immunohistochemical evaluation is essential in distinguishing CNs and IOs. Generally, CNS are immunoreactive for synaptophysin, neuron-specific enolase, and NeuN, but negative for glial fibrillary acidic protein (GFAP), although reactive astrocytes within the lesion are GFAP positive [22]. The neurocytes in CN show low Ki-67 (<2%) immunostaining. IOs are negative for neuronal markers but diffusely positive for GFAP. Because NeuN and GFAP immunohistochemical stains were not available, immunoreactivity for neuron-specific enolase, synaptophysin, and low Ki-67 was sufficient to rule out IO and confirm a diagnosis of CN [22].

Gross total resection (GTR) with complete tumor removal is the treatment of choice for CN, and radiation therapy (RT) is indicated when complete resection is not attained or possible, or in cases of disease recurrence or progression [23, 24]. The median radiation dose in one study was 54 Gy (range, 50–60 Gy) with a median follow-up time of 56 months [24]. The 5-year OS and 5-year PFS were 90% and 76%, respectively [24].

RT remains an important adjuvant treatment that can improve patient survival in the presence of maximal safe resection to levels comparable to those of GTR or GTR + RT. One study analysis suggests that for CN in which total surgical resection is difficult or is located in places where the patient is at risk of developing postoperative neurologic morbidity, MSR + RT can be considered as the next best alternative [23].

A surgical approach is dependent upon the tumor location, operator experience, and preference. Most often, an anterior interhemispheric transcallosal route is used [25]. The use of chemotherapy is not well established and is only usually indicated on the failure of surgery or radiotherapy and or recurrence after initial treatment. Chemotherapy options, as part of multimodal treatment, typically include carmustine, prednisone, vincristine, and cisplatin, although responses to chemotherapy have not been well-characterized [26]. The majority of patients can be expected to be cured of their disease and become long-term survivors.

Generally, CNs have a good prognosis after complete tumor resection with excellent long-term survival (5-year survival 81%) [8], which depends on the size of the tumor and the extent of growth within the ventricular system determining whether it can be completely or incompletely resected [27]. A subset of cases has been termed as atypical and exhibits aggressive behavior. They show atypical histological features including cellular pleomorphism, high mitotic activity, microvascular proliferation, necrosis, and elevated proliferation index (MIB-1 labeling) [28, 29]. The proliferation index as evaluated by immunostaining for MIB-1 staining is under 2.3% in classic neurocytomas but over 5.2% in atypical neurocytomas in one study [30]. In another study, there are 22% of neurocytomas with an MIB-1 labeling index below 2% relapse and 63% of neurocytomas with an MIB-1 labeling index over 2% relapse [31] after tumor resection. Schild et al. [32] report a 5-year local control and survival rate of 100% and 80% after gross total resection without adjuvant therapy in their series of 32 patients with central neurocytomas [32]. Little is mentioned about the prognosis of CN without treatment in the literature search. In our case, the histomorphology showed no atypical histologic features and a proliferative index of <2%.

The diagnosis of CN and IO has been discussed earlier with the presence of pineocytomatous rosettes and immunohistochemical positive staining for neuronal markers such as NSE, NeuN, and SYN favoring a diagnosis of CN. Treatment of IO is by surgical resection; however, because of the infiltrative nature, the tumor is usually poorly delineated from the normal brain parenchyma making complete resection impossible [33]. As a result, resection is usually followed by chemotherapy and radiotherapy. IOs are known to respond well to chemotherapeutic agents and are treated with procarbazine, lomustine, and vincristine (PCV) [34, 35]. Numerous studies have since documented similar results with PCV therapy, with favorable responses seen in about 70% of patients and a median response duration being about 12–18 months [36].

It is essential to differentiate CNs from IOs as the latter is infiltrative making complete resection impossible. In contrast, the former is well-circumscribed and can be easily resected when detected and diagnosed early, with minimal risk of infiltration of the surrounding brain parenchymal leading to distant metastasis. Additionally, CNs are known to have a poor clinical response to chemotherapeutic agents compared to IOs [36].

## 4. Conclusion

CNs are rare, slow-growing low-grade intraventricular neoplasms, and amenable to complete surgical resection commonly. Over time, the enlarging mass within the ventricular system may result in obstructive hydrocephalus leading to increased intracranial pressure and complicated neurologic deficits. We encourage the routine neuroimaging of children and teenagers in our setting, who present with signs and symptoms of increased intracranial pressure. Radiologic imaging, revealing an intraventricular tumor with calcifications, may suggest a CN or IO. Immunohistochemical evaluation with neuronal markers is crucial in characterizing the two in our setting, as both tumors share similar histomorphological features but different prognoses and clinical outcomes.

## Abbreviations

CN: Central neurocytoma
GFAP: Glial fibrillary acidic protein
IO: Intraventricular oligodendroglioma
ICSOL: Intracranial space occupying lesion.
